# A Pilot Study to Assess the Reliability of Sensing Joint Acoustic Emissions of the Wrist

**DOI:** 10.3390/s20154240

**Published:** 2020-07-30

**Authors:** Daniel M. Hochman, Sevda Gharehbaghi, Daniel C. Whittingslow, Omer T. Inan

**Affiliations:** 1 Woodruff School of Mechanical Engineering, Georgia Institute of Technology, Atlanta, GA 30332, USA; 2 School of Electrical and Computer Engineering, Georgia Institute of Technology, Atlanta, GA 30313, USA; sevda@gatech.edu (S.G.); omer.inan@ece.gatech.edu (O.T.I.); 3 Wallace H. Coulter Department of Biomedical Engineering, Georgia Institute of Technology, Atlanta, GA 30332, USA; d.c.whittingslow@emory.edu; 4 School of Medicine, Emory University, Atlanta, GA 30322, USA

**Keywords:** joint acoustic emissions, wearable sensing, wrist joint health

## Abstract

Joint acoustic emission (JAE) sensing has recently proven to be a viable technique for non-invasive quantification indicating knee joint health. In this work, we adapt the acoustic emission sensing method to measure the JAEs of the wrist—another joint commonly affected by injury and degenerative disease. JAEs of seven healthy volunteers were recorded during wrist flexion-extension and rotation with sensitive uniaxial accelerometers placed at eight locations around the wrist. The acoustic data were bandpass filtered (150 Hz–20 kHz). The signal-to-noise ratio (SNR) was used to quantify the strength of the JAE signals in each recording. Then, nine audio features were extracted, and the intraclass correlation coefficient (ICC) (model 3,*k*), coefficients of variability (CVs), and Jensen–Shannon (JS) divergence were calculated to evaluate the interrater repeatability of the signals. We found that SNR ranged from 4.1 to 9.8 dB, intrasession and intersession ICC values ranged from 0.629 to 0.886, CVs ranged from 0.099 to 0.241, and JS divergence ranged from 0.18 to 0.20, demonstrating high JAE repeatability and signal strength at three locations. The volunteer sample size is not large enough to represent JAE analysis of a larger population, but this work will lay a foundation for future work in using wrist JAEs to aid in diagnosis and treatment tracking of musculoskeletal pathologies and injury in wearable systems.

## 1. Introduction

The wrist is one of the most injured joints in athletes, especially in adolescents. Of all adolescents who participate in athletics, 1.3% have sustained wrist injuries via traumatic injuries in contact sports and overuse injuries in golf, racquet sports, and gymnastics [1]. Chronic joint disorders also show high prevalence around the wrist: 34% of children with juvenile idiopathic arthritis (JIA) have active hand and wrist impairments, 15–23% of whom experience active arthritis around the hand and wrist [2]. Further, disabilities of the wrist and hand are the second largest cause of missed workdays [3]. These injuries and chronic joint disorders impact patients’ quality of life and ability to participate in hobbies, athletics, and other activities; these conditions also put pressure on health systems, requiring diagnosis and treatment efforts in a large population of patients [4]. The current standard in noninvasive diagnostic tools for such conditions include a combination of (1) imaging—which is expensive—and (2) physical examination, mobility assessments, and patient-reported pain assessments—all of which are subjective to either the patient or physician. The weaknesses of these tools are compounded when attempting to track treatment progress, as subjective data is weaker than quantitative data when tracking across time, and repeated imaging procedures compound the high costs. Therefore, a technique which allows quantitative measurements on inexpensive hardware would relieve much of the pressure on health systems in diagnosing and tracking treatment for injuries and chronic joint disorders afflicting the wrist.

Recent work has demonstrated the use of inexpensive accelerometers to noninvasively aid in joint health assessment by sensing the vibration on the surface of the skin—termed “vibroarthrographic” signals [5], or more commonly, “joint acoustic emissions” (JAEs) or “joint sounds”—associated with the articulation of the underlying joint [6,7,8,9,10,11,12,13,14,15,16,17,18]. This technique may lead to improved diagnosis and treatment tracking of joint injuries and chronic joint conditions with improved convenience and cost compared to the current standard. However, these works have focused primarily on the knee because of the prevalence of joint injuries and chronic joint disorders afflicting the knee due to the high loads and constant use it sustains. Since the wrist joint is also commonly afflicted with joint injuries and chronic joint disorders, it is necessary to assess the reliability of using JAEs to aid in wrist joint health assessment as explored around the knee [6,7,8,9,10,11,12,13,14,15,16,17,18]. To demonstrate JAE recording reliability, JAEs from healthy volunteers can be used [19]. Many studies focusing on the knee joint selected microphone locations 2 cm lateral and medial to the patellar tendon as the best locations to monitor knee JAEs due to the lower acoustic impedance in the route from the knee joint articulating surfaces to the skin [6]. Another study assessed reliability of recording knee JAEs from the tibial plateau and the top of the patella and found those locations to be highly repeatable within recording sessions but had poor intersession repeatability [19]. Similar to these studies, a location or set of locations known to repeatably record wrist JAEs are needed to monitor the JAEs produced by the wrist and facilitate future design of wearable JAE monitoring systems in a similar manner to other studies which explore sensing modalities at locations around the wrist or knee [14,20]. Previous JAE studies around the knee have also explored prescribing different motions to excite JAEs in different manners. Examples of such motion which have been prescribed in previous studies includes unloaded knee flexion-extension, as well as exercises which introduce higher loads on the knee such as sit-to-stand, squat, stair climbing, and vertical leg press [8,15,16,18,19,21]. A study of JAEs rooting from the wrist joint should also determine what the best exercises are to prescribe to patients for reliably assessing wrist health. This study seeks to use signal strength and repeatability levels [10,19] evaluated using nine audio features to assess JAE recording quality from a set of healthy volunteers performing two different exercises at eight different anatomically and experimentally determined locations around the wrist.

## 2. Materials and Methods

### 2.1. Study Design and Ethics

This study followed the Guidelines for Reporting Reliability and Agreement Studies (GRRAS) [22]. All human volunteers research was conducted in accordance with the Declaration of Helinski (with its recent modification at Fortaleza, 2013) under approval from the Georgia Institute of Technology Institutional Review Board (#H15398). Volunteers provided written informed consent prior to participation in the study.

### 2.2. Participants

Seven healthy college-aged volunteers (three male/four female, 24.9 ± 3.5 years, 65.3 ± 8.4 kg, and 168.0 ± 10.1 cm) were recruited. Inclusion criteria for participation in this study dictated volunteers must have no history of major wrist injury or degenerative joint disease. Additionally, if volunteers had changes to wrist joint health between recording sessions, they would be excluded from the study. No volunteers met this exclusion criteria, so no such exclusions were made. Other factors which may affect wrist JAEs such as volunteers’ daily medication usage, day-to-day wrist activity levels, and history of previous minor wrist injuries were not controlled. An additional volunteer who had juvenile idiopathic arthritis (JIA) as a child (female, 41 years, 75 kg, 175 cm) was included to facilitate proof-of-concept qualitative comparisons to our healthy volunteers, and to allow the development of hypotheses for future studies to address.

A previously recorded dataset (seven male/three female, 25.1 ± 2.9 years, 72.4 ± 13.4 kg, and 170.1 ± 13.4 cm, 5–10 motion cycles each) of JAE recordings from knee squat exercises recorded at sites 2 cm medial and lateral to the patellar tendon on both legs of college-aged individuals with no history or major knee joint injury or degenerative disease was also used in order to compare the signal strength measurements attained in the analysis of the recordings from the wrist against the two locations on the knee known to provide salient knee health assessment. The same data acquisition system, preprocessing steps, and signal strength assessment methods were used in this dataset as were used in our wrist recordings to ensure consistency in methods to provide a valid comparison. 

### 2.3. Experimental Protocol

**Figure 1:**
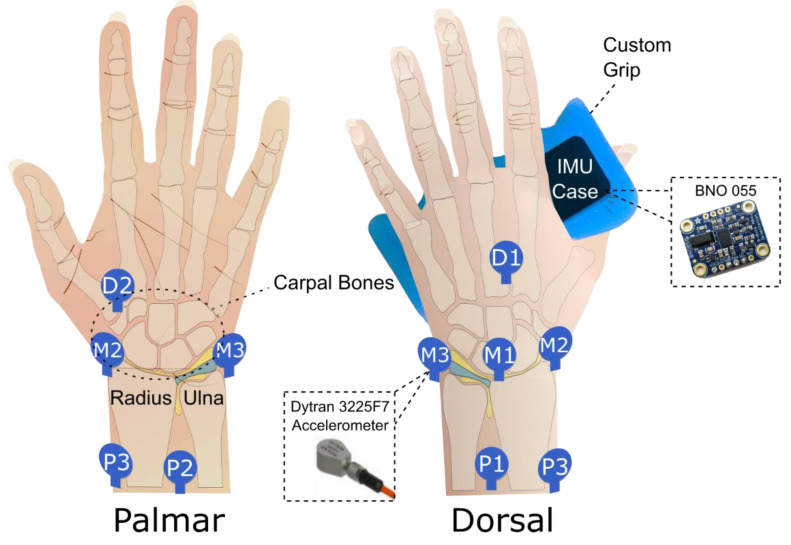
Testing setup for recording joint acoustic emissions (JAEs) from the wrist. During a recording, the wrist has accelerometers (either P1–P3 and D1 or M1–M3) attached to the skin with double sided tape. The volunteer holds the grip which contains the inertial measurement unit (IMU) and an accelerometer to press against the skin at location D2. The data from the four accelerometers were synchronously recorded via a National Instruments data acquisition unit, which was controlled by a computer running MATLAB.

On each day of participation, volunteers preconditioned their wrists by moving them around for 20–30 s. Volunteers then practiced following the animations which guided them to move their wrist according to the two prescribed exercises at the desired movement speed (2 s cycle period). Then, four contact microphones were attached to four of our eight selected locations (shown in Figure 1) around the wrist using double-sided tape. JAEs were then recorded while the volunteers performed 10 cycles of exercises, again following our animations, three times for each combination of unweighted flexion-extension and rotation exercises at two sets of microphone location for both wrists. Volunteers performed this protocol on each of two separate days, separated by less than a week. Descriptions and reasoning for selected exercises, and locations are provided in Appendix A.

### 2.4. Signal Extraction, Signal Processing, and Signal Strength Analysis

Our JAE sensing system consists of four miniature uniaxial accelerometers (Series 3225f7, Dytran Instruments, Inc., Chatsworth, CA, USA) with wide bandwidth (2 Hz–10 kHz), and high sensitivity (100 mV/g). The accelerometers, used as contact microphones, were fixed to the skin using double-sided tape (Elizabeth Craft Designs, Evergreen, CO, USA). The other end of the accelerometers was connected to a computer via a data acquisition system (USB-4432, National Instruments, Austin, TX, USA) recording vibrations at a sampling rate of 50 kHz for subsequent processing using scripts in MATLAB (MathWorks, Natick, MA, USA).

An inertial measurement unit (IMU) (BNO055, Adafruit Industries, New York, NY, USA) was connected to a microcontroller (UNO, Arduino, Somerville, MA, USA) to monitor the motion of the wrist joint relative to the forearm while volunteers performed the wrist exercises necessary to excite JAEs. To best track wrist motion, the forearm was held stationary by strapping it to the arm of the volunteer’s chair and the IMU had to be held in the hand of the wrist in motion. A custom grip (shown in Figure 1) made of silicone gel (Ecoflex Gel Platinum Silicone Gel, Smooth-On, Easton, PA, USA) was developed with slots designed to fit the accelerometers and a custom-developed IMU case, allowing volunteers to hold the IMU and press the accelerometer onto the skin of the palm while also constraining fingers. The combination of the broad bandwidth contact microphones (accelerometers) to pick up JAEs and an IMU to track movement allows us the best chance to reliably monitor JAEs from the wrist in motion.

The first step in processing the raw audio signal was applying a Kaiser window bandpass filter (150 Hz–20 kHz) to the audio signal recordings. At this point, we performed qualitative analysis by visually comparing the filtered waveforms (as is done in Section 4.1) and listening to the sounds recorded by the microphones of all healthy volunteers who had no history of major wrist injury or illness to the recordings from the volunteer who has a history of JIA from childhood and audible wrist JAEs. Once the presence of JAEs was confirmed in our dataset of healthy volunteers, we segmented the microphone signal of our dataset into the 10 movement cycles of prescribed motion using the IMU signal. Each cycle was windowed into 400 ms long frames with 50% overlap, and nine audio features (zero-crossing rate, acoustic energy, spectral centroid, spectral spread, spectral flux, harmonic ratio, spectral crest, spectral decrease, and spectral slope) were extracted and obtained in the statistical analysis. The selection reasoning for these raters is described in Appendix A. The measurements and raters in this experiment were not calculated independently.

To calculate the signal-to-noise ratio (SNR), the Teager energy operator was used to find the characteristic JAE clicks in the signal, chosen as the peaks greater than 20% of the range of the signal value as has been proven to be successful in JAE analysis by Semiz et al. [9]. Click windows were extracted as the time ± 50 ms of each detected click. The sections of the recording not containing clicks were used as the windows of noise and motion artifacts. The power of each window was calculated, and the ratio of power of the click window was taken against the power of the motion artifact window to yield an SNR for each recording. These can then be summarized by location to assess the contribution of the motion artifacts at each microphone placement location and for each exercise.

### 2.5. Statistical Analysis

All statistical analysis was performed using scripts in MATLAB. The mean, median, and standard deviation of the features of all windows were calculated and stored as a vector of features for each cycle, where there are 60 such feature vectors for each combination of volunteer, wrist, exercise, and location (six sessions and 10 cycles for each). The average of each feature over those 60 vectors was calculated to give a single averaged feature vector for each combination of volunteer, wrist, exercise, and location. To calculate the intraclass correlation coefficient (ICC) to describe intersession reliability, each feature vector acts as a measurement where every feature is a rater. We used the two-way mixed effects, consistency, multiple raters/measurements model of ICC (model 3,*k*) to calculate ICC values with a 95% confidence interval (CI) [23]. All measurements at each location were used to calculate the ICC for each location. Likewise, all measurements for each exercise were used to calculate the ICC for the two exercises. Intrasession reliability was calculated using the same ICC calculation methods as for intersession reliability, where the two separate days of recordings for each volunteer represented different measurements; thus, there are twice as many feature vector measurements for ICC calculations. Our ICC values were assessed according to Fleiss [24], where values less than 0.40 are “poor”, values between 0.40 and 0.75 are “fair to good”, and values greater than 0.75 are “excellent”. We presented these values at a high level among all volunteers and calculated those on a volunteer-by-volunteer basis. In addition, to assess the volunteer variability, the standard deviation between volunteers at each location was reported. 

To find the JS divergence, distributions of each feature are needed: the nine features for each bin were kept such that they could be summarized as a histogram of the feature distribution for each recording of 10 cycles. Then, the average distribution of similar recordings on a day (all recordings from the same session date, volunteer, wrist, exercise, and location) was taken. The KL divergence of each of those similar recordings was calculated using the averaged distribution as the “ground truth recording” [25,26]. Averaging these KL divergence values gave the intrasession JS divergence of each feature for that combination of session date, volunteer, wrist, exercise, and location. JS divergence was then averaged by location and exercise [2]. Justifications for data preprocessing steps, feature selection, and measurements of reliability and signal strength are contained in Appendix A.

The standard error of measurement (SEm) was found for both JS divergence and SNR calculations. The SEm was the multiplied by 1.96 to give a confidence bound, which was added to the top and bottom of the measurement (JS Divergence or SNR) to give a 95% CI. The coefficient of variation (CV) was also calculated for each feature at each location and exercise to assess intersession and intrasession variability, where low levels of variation are defined as CV values less than 12% [19].

## 3. Results

### 3.1. Reliability Measurements

The calculated intrasession ICC values with 95% CIs for flexion-extension and rotation exercises over all tested locations are displayed in Table 1. Intersession ICCs with 95% CIs for flexion-extension and rotation exercises over all tested locations are displayed in Table 2. Standard deviation of intrasession ICC between volunteers was 0.082 for both exercises, and standard deviation of intersession ICC between volunteers was 0.091 for both exercises. Repeatability analysis using intrasession JS divergence (values closer to 0 indicate high levels of similarity) on the nine identified features extracted from the filtered acoustic signal gave median JS divergence of 0.190 (95% CI of 0.186–0.193) for flexion-extension and 0.187 (95% CI of 0.184–0.190) for rotation. Assessing the variation of feature values for each exercise yielded mean CV values displayed in Table 1 and Table 2. 

Intrasession ICC with 95% CIs for all locations are shown in Table 1. The intersession ICC with 95% CIs for all locations are shown in Table 2. The standard deviation of intrasession ICC across volunteers was 0.078 at location P1, 0.040 at location P2, and between 0.001 and 0.009 for locations P3, D1, D2, and M1–M3, and the standard deviation of intersession ICC across volunteers was 0.116 at location P1, 0.025 at location P2, and between 0.002 and 0.005 for locations P3, D1, D2, and M1–M3. Performing similar repeatability analysis using intrasession JS divergence on these locations gave median values between 0.18 and 0.20 (with 95% CIs in the same range). Assessing the variation of feature values for each location yielded mean CV values displayed in Table 1 and Table 2.

### 3.2. Signal Strength

Assessing the signal strength relative to noise level after filtering, the median SNR was found to be 6.0 dB (95% CI of 5.8–6.2 dB) for flexion-extension and 6.1 dB (95% CI of 5.7–6.5 dB) for rotation with standard deviation between volunteers of 1.9 dB for flexion-extension and 1.0 dB for rotation. 

**Figure 2:**
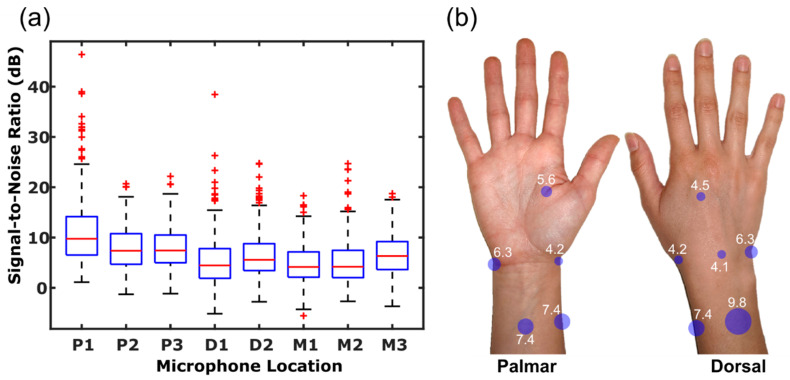
(**a**) Box-and-whisker plot of signal-to-noise ratios at each microphone placement location filtered using a Kaiser-window bandpass filter with a passband of 150 Hz–20 kHz. (**b**) Signal-to-noise ratio is displayed as circles shown at the microphone location that signal-to-noise ratio (SNR) was measured at. SNR magnitude is represented as the radius of the circle and median SNR (dB) values are displayed next to the circles.

SNR values for each recording and summarizing by location yields Figure 2. Here, it can be demonstrated that over all volunteers, locations P1–P3 had higher median SNRs (9.8 with 95% CI of 8.6–10.9, 7.4 with 95% CI of 6.7–8.0, and 7.4 with 95% CI of 6.8–8.0 dB, respectively) than other locations (*p* < 0.001, using two-sample *t*-test with Bonferroni correction). Locations D1, D2, and M1–M3, where median SNRs were 4.5 (95% CI of 3.6–5.3), 5.6 (95% CI of 4.9–6.3), 4.1 (95% CI of 3.6–4.7), 4.2 (95% CI of 3.5–4.9), and 6.3 (95% CI of 5.7–6.9) dB, respectively. Additionally, the standard deviation of SNRs among volunteers ranged from 1.6 to 2.5 dB. The dataset of recordings from knee squats of healthy volunteers showed a median SNR of 5.5 (95% CI of 4.8–6.3) dB at 2 cm medial to the patellar tendon and a median SNR of 7.2 (95% CI of 6.6–7.9) dB 2 cm lateral to the patellar tendon.

## 4. Discussion

### 4.1. Evaluating the Ability of Prescribed Exercises to Excite Joint Sounds

**Figure 3:**
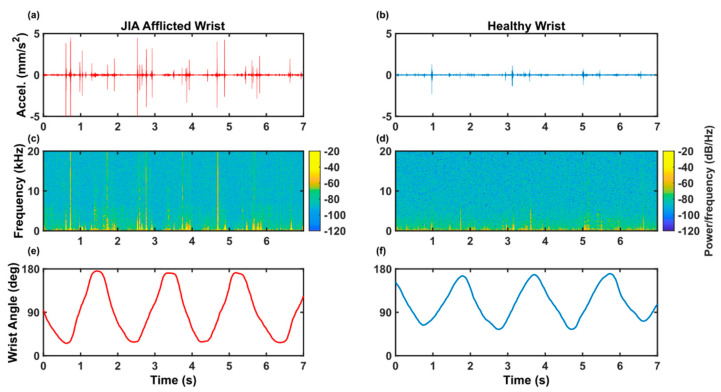
(**a**,**c**,**e**) Volunteer who was afflicted with juvenile idiopathic arthritis (JIA) as a child and continued to have audible JAEs from the wrist during articulation. (**b**,**d**,**f**) Volunteer with no history of wrist pathology. (**a**,**b**) Time domain of acoustic signal from the wrist. (**c**,**d**) Spectrogram of the acoustic signal displayed above it. (**e**,**f**) Motion data from the IMU recorded synchronously with the acoustic signal.

Recordings taken from the wrist joint during the prescribed motions must capture wrist JAEs. The highest quality JAE signals gathered in previous work come from Whittingslow et al.’s study on joint sounds within a cadaver knee, as observed JAEs did not exist in a knee before meniscus tear and meniscectomy. Thus, they could be directly attributed to changes within the articulating surfaces within the knee [27]. For our study, we qualitatively assessed JAEs recorded from an additional volunteer who was afflicted with JIA as a child and has wrist acoustic emissions which can be detected audibly. These recordings, shown in Figure 3, closely resemble the grinding and clicking characteristic of JAEs from Whittingslow et al.’s study as well as the JAEs described in earlier studies [7,8,9,27] suggesting that our protocol records JAEs from the wrist during both flexion-extension and rotation which are similar to those which have been able to provide diagnostic power in previous studies on the knee. JAEs from healthy volunteers were less frequent than JAEs from the volunteer with a history of JIA but were often periodic with the same period as the prescribed wrist motions (2 s), as is expected in JAE recordings from healthy volunteers [6,28].

Since both prescribed exercises excite wrist JAEs, we can use JS divergence and intersession and intrasession ICC and CV to assess the repeatability of these exercises exciting wrist JAEs [10,19]. Our results indicate fair levels of intrasession and intersession repeatability in exciting JAEs with unweighted flexion-extension, and high levels of intrasession and intersession repeatability in recording JAEs from a wrist in rotation, with low variation between volunteers. The difference in repeatability in these exercises has moderate significance (*p* < 0.05 for intersession ICC and *p* < 0.1 for intrasession ICC, using two-sample *t*-test with Bonferroni correction), so we may conclude that rotation exercises more reliably create the dynamic interactions within the wrist joint which excite JAEs. Similar repeatability analysis using intrasession JS divergence (values closer to 0 indicate high levels of similarity) on the nine identified features extracted from the filtered acoustic signal confirms the high levels of repeatability also demonstrated by the ICCs. Both exercises showed acceptable levels of signal strength with some variation between the healthy volunteers, but there was not a significantly better exercise for minimizing motion artifacts (*p* = 0.30, using paired sample *t*-test with Bonferroni correction). However, SNR may be more dependent on microphone location due to motion artifacts, thus eliminating noisier locations would improve the overall performance of each exercise. We determined both unweighted flexion-extension and rotation exercises can repeatably excite and record wrist JAEs with moderate signal strength, and the features of sounds which have performed well in knee joint health classification studies show high repeatability in our recordings. Therefore, we can conclude that both unweighted wrist flexion-extension and rotation exercises are suitable for use in clinical studies in future work. 

### 4.2. Evaluating Microphone Placement Locations around the Wrist

It has been demonstrated that both wrist flexion-extension and rotation exercises consistently excite wrist JAEs which can be picked up by contact microphones placed on the skin. However, some microphone placement locations have higher levels of noise due to motion artifacts. Quantifying this as an SNR value for each recording and summarizing by location yields the results displayed in Figure 2, which indicates that locations P1–P3 gave the highest SNR values (*p* < 0.001, using two-sample *t*-test with Bonferroni correction), and locations D2 and M3 showed moderately higher signal strength than the rest of the locations (*p* < 0.001, using two-sample *t*-test with Bonferroni correction). Additionally, the standard deviation of SNRs among volunteers indicates some variability in noise levels at different locations among volunteers. Though this may alter which location produces the best signal quality for individual volunteers, the overall separation between proximal locations and all other locations indicates that due to the decreased skin motion relative to the underlying skeletal structure [29,30], these locations often minimize the detrimental impact of motion artifacts on JAE recordings. When performing the same analysis to estimate SNR from a dataset of recordings from knee squats of healthy volunteers, the values were similar to the SNR values gathered around the wrist, indicating that recordings from the wrist have similar signal quality to those which have been correlated to joint health around the knee.

In our chosen feature set both within and between single-day recording sessions, we saw intrasession and intersession ICC values demonstrated excellent repeatability in picking up JAEs at all locations except P1 and P2, which themselves had fair-to-good repeatability levels. Additionally, the standard deviation of ICC values across volunteers indicated low levels of variability between volunteers. Furthermore, our feature set showed moderate-to-low intrasession variability and moderate intersession variability. These repeatability findings compare favorably against a similar study from Kalo et al. on knee JAE repeatability which found intrasession ICCs of the median power frequency ranging from 0.85 to 0.95 at the tibia and 0.73 to 0.87 at the patella and intersession ICCs from 0.24 to 0.33 at the tibia and 0 to 0.82 at the patella [19], indicating our wrist recordings picked up JAEs with similar or better repeatability levels than similar knee recordings in healthy volunteers at most locations. There is only enough separation to conclude any statistical significance in location reliability based on intrasession ICC between the lowest performing location (P1) and the group of highest performing locations (D2 and M1–M3) (*p* < 0.05 for intrasession ICC, using two-sample *t*-test with Bonferroni correction), and separation decreases so good separation (*p* < 0.05, using two-sample *t*-test with Bonferroni correction) is only present between the lowest and highest (M3) performing locations for intersession ICC. Additionally, our locations with highest repeatability also showed highest level of noise and motion artifact interference, which we have observed to inflate our consistency measurements, further reducing the true separation between these measures across locations. However, the degree of repeatability shown by the ICC (using measures which have been effective in knee JAE health classification [7,8,21,27]) demonstrates any of these locations will consistently record high quality JAEs [10,19]. 

The combination of fair-to-high repeatability demonstrated by ICC and JS divergence analysis at all locations around the wrist and the acceptable signal strength demonstrated with the SNR at locations P1–P3, D2, and M3 allows us to conclude that locations P1–P3, D2, and M3 are suitable for high quality repeatable wrist JAE recordings. The strong SNR values at locations P1–P3 had high separation over all other locations (*p* < 0.001, using two-sample *t*-test with Bonferroni correction), which had relatively weaker SNR scores. Additionally, the strongest signal strength demonstrated at location P1 showed high levels of separation from all other locations (*p* < 0.001, using two-sample *t*-test with Bonferroni correction). This reveals the best locations for minimizing artifact interference while still having fair repeatability are proximal to the wrist joint, and location P1, which is 3 cm proximal to the wrist joint and centered between the radius and ulna on the dorsal side, is best for minimizing noise and motion artifact levels. On the other hand, locations P1 and P2 have the lowest levels of intersession and intrasession repeatability, which can be attributed to reduced proximity to the wrist joint and the fact that the soft tissue these locations rest upon will not transmit vibrations as well as the harder tissues of other locations, such as locations P3, D1, D2, and M1–M3, which are all clustered with high repeatability levels. The best balance of moderate-to-high signal strength and excellent repeatability of JAE recordings in healthy volunteers can be shown at locations P3, D2, and M3. 

### 4.3. Limitations and Future Work

Our volunteer sample size (*n* = 7) and the low-to-medium levels of variability between volunteers for our measurements means we cannot know if our sample of healthy volunteers is representative of a larger population with varied wrist joint pathologies. Future studies researching the effectiveness of quantifying wrist joint health using the locations described in this study must first perform qualitative assessments by looking at and listening to the audio signal to identify grinding and clicking sounds characteristic of JAEs to confirm the quality of wrist JAE recordings. 

Locations P1–P3, D2, and M3 around the wrist have JAE recording quality and reliability levels during unweighted flexion-extension and rotation which compare well against similar measures around the knee during sit-to-stand and squats. These wrist locations and exercises can be employed in future studies aiming to show whether wrist JAEs demonstrate similar levels of diagnostic and treatment-tracking power for wrist injuries and degenerative diseases as previous studies centered around the knee presented [7,8,9,10,11,12,14,16,17,27]. Additionally, different form factors can be explored to make affordable wrist JAE monitoring technology accessible to health systems, or even to patients for wearable at-home joint health assessment [17]. 

## 5. Conclusions

This work used recordings from a set of healthy volunteers with no history of wrist joint injury or degenerative disease, and one adult volunteer who had a history of JIA from childhood, to provide a framework with which wrist JAEs can be compared. We validate that this framework will consistently excite and record high quality JAEs through confirmation of the ability of the prescribed exercises and microphone locations to excite and record JAEs from the wrist, assessing the impact of noise and motion artifacts, and performing repeatability testing on our measurements of raters that have demonstrated importance to knee joint health classification based on JAEs. Both flexion-extension and rotation exercises are suitable to reliably excite JAEs from the wrist joint with high signal strength, and all eight tested microphone locations show fair-to-high levels of repeatability similar to recordings of the knee from the top of the tibia and patella. The locations proximal to the end of the radius also demonstrated higher signal strength through the SNR than other locations around the wrist and the locations tested around the knee, whereas locations P3, D2, and M3 showed a balance of good signal strength and excellent repeatability. The combination of these exercises and microphone locations provides the framework with which future work may be able to use wrist JAEs to aid in diagnosis and monitoring of patient populations with chronic joint conditions (such as JIA) or wrist injuries as has been done in previous studies on the knee. This framework could be used to create techniques for clinical use of wrist JAEs, which may allow for the development of wearables for at-home wrist joint health monitoring. Such technology would allow for quicker diagnosis of wrist joint injuries and chronic joint conditions, and could improve treatment monitoring by providing clinicians with quantitative measurements to help assess patients’ wrist joint health more frequently without the need for additional clinic visits, which would benefit patient outcomes while reducing the burden on the medical systems that treat such injuries and chronic joint conditions. Moreover, given the increased reliance on telemedicine during the current pandemic, technologies for enabling joint health assessment in the home environment are imminently needed to facilitate comprehensive physical examination of joint health in a remote setting. 

## Figures and Tables

**Table 1:** Intrasession intraclass correlation coefficients (ICC) values with 95% confidence intervals and coefficient of variation (CV) evaluated for all tested exercises and at all tested locations around the wrist.

	Intrasession Reliability			
		95% CI		
	ICC	Lower Bound	Upper Bound	CV
**Flexion-Extension**	0.632	0.478	0.758	0.161
**Rotation**	0.820	0.745	0.881	0.107
**Location P1**	0.631	0.477	0.757	0.156
**Location P2**	0.752	0.649	0.836	0.152
**Location P3**	0.847	0.784	0.899	0.153
**Location D1**	0.811	0.734	0.875	0.157
**Location D2**	0.837	0.770	0.892	0.143
**Location M1**	0.849	0.787	0.900	0.099
**Location M2**	0.857	0.798	0.905	0.101
**Location M3**	0.872	0.819	0.915	0.109

**Table 2:** Intersession ICC values with 95% confidence intervals and CV evaluated for all tested exercises and at all tested locations around the wrist.

	Intersession Reliability			
		95% CI		
	ICC	Lower Bound	Upper Bound	CV
**Flexion-Extension**	0.631	0.525	0.723	0.236
**Rotation**	0.789	0.729	0.841	0.183
**Location P1**	0.629	0.399	0.801	0.225
**Location P2**	0.760	0.614	0.871	0.241
**Location P3**	0.847	0.754	0.917	0.233
**Location D1**	0.817	0.706	0.902	0.231
**Location D2**	0.840	0.743	0.914	0.232
**Location M1**	0.855	0.768	0.922	0.169
**Location M2**	0.870	0.791	0.930	0.170
**Location M3**	0.886	0.817	0.938	0.176

## Appendix A

In previous studies on the knee, unloaded flexion-extension exercises produced JAEs with meaningful information related to the function and pathologies of the joint [6,7,8,9,10,11,12,13,14,15,16,17,18]. The wrist is an ellipsoid joint with movement in more than two planes [31], where flexion-extension, radial-ulnar deviation, and circumduction (rotation) may create dynamic interactions within the intercarpal joints to elicit JAEs. Flexion-extension and rotation were tested in this study. Radial-ulnar deviation was eliminated in the preliminary experiments of our study, as it could not consistently excite JAEs from the wrist in healthy volunteers. The addition of weights was also considered due to their ability to excite more JAEs, as shown in previous works [15]. However, the main goal of this investigation is to determine if JAEs can consistently be monitored around the wrist, and Jeong et al. found adding weights reduced the consistency of JAEs in the knee [15]. Our preliminary experiments also found a reduced level of consistency in JAEs coming from the wrist when adding weights; thus, weighted conditions were excluded. This leaves unweighted flexion-extension and rotation as the prescribed motions, where each exercise is performed with a cycle duration of 2 s due to the consistency of the wrist JAEs gathered at that motion frequency during preliminary tests.

The wrist joint is described as a curved line between the styloid processes of the radius and ulna, curving proximally 1 cm [32]. The three proximal microphone locations (P1–P3) are 3 cm proximal to the wrist joint with the first centered between the radius and ulna on the dorsal side, the second centered between the radius and ulna on the volar (palmar) side, and the third on the skin covering the radius. The first and second locations were selected because of the relatively sparse soft tissue in the region allowing for a relatively unobstructed path for vibration to propagate from the wrist, whereas the third location allows for sound conduction along the radius [33]. Additionally, these locations are optimal locations for a wristwatch-style wearable design and have minimal skin motion relative to the underlying skeletal structure [29,30], which should minimize motion artifacts. The distal microphone locations are 3 cm distal to the wrist joint, where the first (D1) is centered between the second and third metacarpal bones on the dorsal side, and the second (D2) is centered between the first and second metacarpal bones on the palmar side. These were selected for proximity to the distal end of the carpal bones, the limited soft tissue for JAEs to travel unobstructed [33], and small amount of skin motion relative to the underlying bone structure [29,30]. D2 also allows us to listen to wrist JAEs through the custom-designed grip, a potential design for an at-home joint health monitoring system. The middle three microphone locations (M1–M3) are all on the wrist joint, where M1 is 1 cm distal to the dorsal tubercle, M2 is distal and adjacent to the radial styloid process, and M3 distal and adjacent to the ulnar styloid process. These locations were selected for their location on the wrist joint with a soft tissue pathway for JAE propagation [33]. Skin motion is high in these locations relative to the underlying skeletal structure [29,30]. Thus, motion artifacts within the signals, characterized as high signal power without the grinding or clicking sounds characteristic of JAEs, are expected. Other possible locations were not included either to minimize redundancy or because of poor performance in recording the characteristic clicking of JAEs during preliminary testing. The data acquisition unit used in this experiment has four input channels, and eight locations were selected for microphone placement experiments, so these locations were split into two sets of locations which were recorded separately.

To best quantify JAE signal strength and repeatability, recordings must first be filtered. Previous work in the analysis of JAEs from the knee joint has concluded that JAEs have most of their frequency components between 20 Hz and 20 kHz [6]. Frequency analysis of several intentionally generated and recorded motion artifacts such as skin and finger motion reveal the presence of strong frequency components up to 100 Hz, which is undesired. Additionally, wrist rotations are much more affected by the stiffness of the wrist than the inertia of the wrist [12], which increases the frequency of the natural frequencies emitted by the wrist. Thus, a Kaiser-window bandpass filter (150 Hz–20 kHz) should minimize the unwanted noise of these low-frequency motion artifacts and noise inherent to the system as much as possible while maximizing the signal quality of the frequency band of JAEs which have been proven to provide strong joint health assessment capability [7,8].

Once the audio signal recorded from the microphones was filtered, cycles were extracted using IMU signal. Then, each cycle was windowed into 400 ms long frames with 50% overlap, similar to the windowing done by Whittingslow et al. in studies on volunteers with JIA [8]. We then extract distinct features of sound which are known to relate to knee JAEs need to be calculated. Based on a review of previous studies which were able to use knee JAEs to help in diagnostic end goals [7,8,9,11,12,14,16,17,21,27], nine audio features were selected as the most important features to describe JAEs: zero-crossing rate, acoustic energy, spectral centroid, spectral spread, spectral flux, harmonic ratio, spectral crest, spectral decrease, and spectral slope.

To quantify the repeatability of each exercise and each microphone location, two similarity measurements were employed: intraclass correlation coefficient (ICC) and Jensen–Shannon (JS) divergence. ICC is a widely used metric of measurement reliability across different raters [23] and has been used successfully in previous JAE repeatability tests [6,18,19]. We also used the JS divergence, a symmetrical variant of the Kullback–Leibler (KL) divergence, which measures the entropy between two probability distributions that are meant to represent the same dataset, to confirm the measurement consistency [25,26]. To assess the effects of the unwanted noise and artifacts in each location, the signal-to-noise ratio (SNR) was calculated.

## References

[1] Rettig A.C. (1998). Epidemiology of hand and wrist injuries in sports. Clin. Sports Med..

[2] Hoeksma A.F., Zinger W.G., Van Rossum M.A., Dolman K.M., Dekker J., Roorda L.D. (2013). THU0297 High prevalence of hand and wrist impairments in juvenile idiopathic arthritis (JIA). Ann. Rheum. Dis..

[3] Berger R.A., Garcia-Elias M., An K.N., Berger R.A., Cooney W.P. (1991). General Anatomy of the Wrist. Biomechanics of the Wrist Joint.

[4] Woolf A.D., Åkesson K. (2001). Understanding the burden of musculoskeletal conditions: The burden is huge and not reflected in national health priorities. BMJ Br. Med. J..

[5] Blodgett W.E. (1902). Auscultation of the Knee Joint. Bost. Med. Surg. J..

[6] Teague C.N., Hersek S., Toreyin H., Millard-Stafford M.L., Jones M.L., Kogler G.F., Sawka M.N., Inan O.T. (2016). Novel methods for sensing acoustical emissions from the knee for wearable joint health assessment. IEEE Trans. Biomed. Eng..

[7] Semiz B., Hersek S., Whittingslow D.C., Ponder L.A., Prahalad S., Inan O.T. (2018). Using Knee Acoustical Emissions for Sensing Joint Health in Patients with Juvenile Idiopathic Arthritis: A Pilot Study. IEEE Sens. J..

[8] Whittingslow D.C. (2019). Anatomy of a Joint Sound – Using Joint Acoustic Emissions to Diagnose and Grade Musculoskeletal Disease and Injury. Ph.D. Thesis.

[9] Semiz B., Hersek S., Whittingslow D.C., Ponder L., Prahalad S., Inan O.T. Change point detection in knee acoustic emissions using the teager operator: A preliminary study in patients with juvenile idiopathic arthritis. Proceedings of the 2019 IEEE EMBS International Conference on Biomedical and Health Informatics.

[10] Hersek S., Pouyan M.B., Teague C.N., Sawka M.N., Millard-Stafford M.L., Kogler G.F., Wolkoff P., Inan O.T. (2018). Acoustical emission analysis by unsupervised graph mining: A novel biomarker of knee health status. IEEE Trans. Biomed. Eng..

[11] Frank C.B., Rangayyan R.M., Bell G.D. (1990). Analysis of knee joint sound signals for non-invasive diagnosis of cartilage pathology. IEEE Eng. Med. Biol. Mag..

[12] Befrui N., Elsner J., Flesser A., Huvanandana J., Jarrousse O., Le T.N., Müller M., Schulze W.H.W., Taing S., Weidert S. (2018). Vibroarthrography for early detection of knee osteoarthritis using normalized frequency features. Med. Biol. Eng. Comput..

[13] Khan T.I., Kusumoto M., Nakamura Y., Ide S., Yoshimura T. Acoustic emission technique as an adaptive biomarker in integrity analysis of knee joint. Proceedings of the Regional Conference on Acoustics and Vibration 2017.

[14] Ota S., Ando A., Tozawa Y., Nakamura T., Okamoto S., Sakai T., Hase K. (2016). Preliminary study of optimal measurement location on vibroarthrography for classification of patients with knee osteoarthritis. J. Phys. Ther. Sci..

[15] Jeong H.-K., Pouyan M.B., Whittingslow D.C., Ganti V., Inan O.T. (2018). Quantifying the Effects of Increasing Mechanical Stress on Knee Acoustical Emissions Using Unsupervised Graph Mining. IEEE Trans. Neural Syst. Rehabil. Eng..

[16] Madeleine P., Andersen R.E., Larsen J.B., Arendt-Nielsen L., Samani A. (2020). Wireless multichannel vibroarthrographic recordings for the assessment of knee osteoarthritis during three activities of daily living. Clin. Biomech..

[17] Athavale Y., Krishnan S. (2020). A telehealth system framework for assessing knee-joint conditions using vibroarthrographic signals. Biomed. Signal. Process. Control..

[18] Bolus N.B., Jeong H.K., Whittingslow D.C., Inan O.T. (2019). A glove-based form factor for collecting joint acoustic emissions: Design and validation. Sensors.

[19] Kalo K., Niederer D., Sus R., Sohrabi K., Groß V., Vogt L. (2020). Reliability of Vibroarthrography to Assess Knee Joint Sounds in Motion. Sensors.

[20] Zhang C., Bedri A.K., Reyes G., Bercik B., Inan O.T., Starner T.E., Abowd G.D. TapSkin: Recognizing on-skin input for smartwatches. Proceedings of the 2016 ACM International Conference on Interactive Surfaces and Spaces: Nature Meets Interactive Surfaces.

[21] Gharehbaghi S., Whittingslow D.C., Ponder L.A., Prahalad S., Inan O.T. Joint Acoustic Emissions as a Biomarker for Knee Health Assessment in Loaded and Unloaded Exercises. Proceedings of the American Society of Biomechanics Annual Meeting 2020.

[22] Kottner J., Audigé L., Brorson S., Donner A., Gajewski B.J., Hróbjartsson A., Roberts C., Shoukri M., Streiner D.L. (2011). Guidelines for Reporting Reliability and Agreement Studies (GRRAS) were proposed. J. Clin. Epidemiol..

[23] Koo T.K., Li M.Y. (2016). A Guideline of Selecting and Reporting Intraclass Correlation Coefficients for Reliability Research. J. Chiropr. Med..

[24] Fleiss J.L., Wiley Classics Library (1999). The Design and Analysis of Clinical Experiments.

[25] Pérez-Cruz F. Kullback-leibler divergence estimation of continuous distributions. Proceedings of the IEEE International Symposium on Information Theory.

[26] Fuglede B., Topsøe F. Jensen-Shannon divergence and Hubert space embedding. Proceedings of the IEEE International Symposium on Information Theory.

[27] Whittingslow D.C., Jeong H.K., Ganti V.G., Kirkpatrick N.J., Kogler G.F., Inan O.T. (2020). Acoustic Emissions as a Non-invasive Biomarker of the Structural Health of the Knee. Ann. Biomed. Eng..

[28] Wiens A.D., Prahalad S., Inan O.T. VibroCV: A computer vision-based vibroarthrography platform with possible application to Juvenile idiopathic arthritis. Proceedings of the Annual International Conference of the IEEE Engineering in Medicine and Biology Society.

[29] Ryu J.H., Miyata N., Kouchi M., Mochimaru M., Lee K.H. (2006). Analysis of skin movement with respect to flexional bone motion using MR images of a hand. J. Biomech..

[30] Richard R., Ford J., Miller S.F., Staley M. (1994). Photographic measurement of volar forearm skin movement with wrist extension: The influence of elbow position. J. Burn Care Rehabil..

[31] Charles S.K., Hogan N. (2011). Dynamics of wrist rotations. J. Biomech..

[32] Surface Markings of the Upper Extremity-Human Anatomy. https://theodora.com/anatomy/surface_markings_of_the_upper_extremity.html.

[33] Fam A.G., Lawry G.V., Kreder H.J. (2016). Musculoskeletal Examination and Joint Injection Techniques.

